# Photosynthesis in newly developed leaves of heat-tolerant wheat acclimates to long-term nocturnal warming

**DOI:** 10.1093/jxb/erad437

**Published:** 2023-11-04

**Authors:** Onoriode Coast, Andrew P Scafaro, Helen Bramley, Nicolas L Taylor, Owen K Atkin

**Affiliations:** ARC Centre of Excellence in Plant Energy Biology, Research School of Biology, The Australian National University, Canberra, ACT 2601, Australia; Natural Resources Institute, University of Greenwich, Central Avenue, Chatham Maritime, Kent ME4 4TB, UK; School of Environmental and Rural Sciences, Faculty of Science, Agriculture, Business, and Law, University of New England, Armidale, NSW 2351, Australia; ARC Centre of Excellence in Plant Energy Biology, Research School of Biology, The Australian National University, Canberra, ACT 2601, Australia; Division of Plant Science, Research School of Biology, The Australian National University, Canberra, ACT 2601, Australia; School of Life and Environmental Sciences, Plant Breeding Institute, Sydney Institute of Agriculture, The University of Sydney, Narrabri, NSW 2390, Australia; ARC Centre of Excellence in Plant Energy Biology, School of Molecular Sciences and Institute of Agriculture, The University of Western Australia, Crawley, WA 6009, Australia; ARC Centre of Excellence in Plant Energy Biology, Research School of Biology, The Australian National University, Canberra, ACT 2601, Australia; Division of Plant Science, Research School of Biology, The Australian National University, Canberra, ACT 2601, Australia; Lancaster University, UK

**Keywords:** Acclimation, electron transport thermotolerance, high night temperature, photosynthetic efficiency, *Triticum aestivum*

## Abstract

We examined photosynthetic traits of pre-existing and newly developed flag leaves of four wheat genotypes grown in controlled-environment experiments. In newly developed leaves, acclimation of the maximum rate of net CO_2_ assimilation (*A*_n_) to warm nights (i.e. increased *A*_n_) was associated with increased capacity of Rubisco carboxylation and photosynthetic electron transport, with Rubisco activation state probably contributing to increased Rubisco activity. Metabolite profiling linked acclimation of *A*_n_ to greater accumulation of monosaccharides and saturated fatty acids in leaves; these changes suggest roles for osmotic adjustment of leaf turgor pressure and maintenance of cell membrane integrity. By contrast, where *A*_n_ decreased under warm nights, the decline was related to lower stomatal conductance and rates of photosynthetic electron transport. Decreases in *A*_n_ occurred despite higher basal PSII thermal stability in all genotypes exposed to warm nights: *T*_crit_ of 45–46.5 °C in non-acclimated versus 43.8–45 °C in acclimated leaves. Pre-existing leaves showed no change in *A*_n_–temperature response curves, except for an elite heat-tolerant genotype. These findings illustrate the impact of night-time warming on the ability of wheat plants to photosynthesize during the day, thereby contributing to explain the impact of global warming on crop productivity.

## Teaser

Wheat photosynthesis and acclimation capacity are directly affected by night warming, despite the temporal separation of night warming from photosynthesis, with varying responses between newly developed and pre-existing leaves.

## Introduction

Wheat (*Triticum aestivum* L) is the most widely cultivated crop, with >220 Mha cultivated worldwide. Wheat yield must increase by 0.83% per annum to meet the projected 44% increase in demand for it by 2050 (CGIAR Advisory Services, 2020). To achieve an environmentally sustainable increase in production of this magnitude, while also coping with rising global surface temperatures, represents a major challenge. Moreover, most research has focused on effects of elevated temperatures during the day on wheat, but rising temperatures have been more pronounced for daily minimums (i.e. night warming) than for daily maximums (Easterling *et al.*, 1997; Alexander *et al.*, 2006; Sillmann *et al.*, 2013). Night warming is linked with a reduction in wheat yield in many regions including the USA, Mexico, and Argentina (Lobell *et al.*, 2005; Prasad *et al.*, 2008; García *et al.*, 2015, 2016, 2018; Russell and Van Sanford, 2020; Fisher *et al.*, 2022). Given the importance of wheat for global food production, it is vital that we develop a more complete understanding of the physiological basis for why nocturnal warming reduces wheat yields. Reflecting this, interest in wheat physiological responses to warm nights has increased, with reports on steady-state photosynthesis, acclimation of leaf and root respiration, and altered source–sink capacity at anthesis and grain filling (Fan *et al.*, 2015, 2017; Impa *et al.*, 2019, 2020; Bahuguna *et al.*, 2022; Posch *et al.*, 2022a). However, there remain key gaps in knowledge, especially in relation to the effect of warm nights on overall photosynthetic capacity, and whether key photosynthetic processes such as the critical temperature above which incipient damage to photosystem II (PSII) occurs (*T*_crit_) acclimate to warm nights. PSII is considered the most heat-sensitive component of electron transport in the photosynthetic machinery, with heat stress resulting in the unfolding of protein complexes and loss of manganese from the oxygen-evolving complex (Enami *et al.*, 1994).

According to the Farquhar, von Caemmerer, and Berry (FvCB model; Farquhar *et al.*, 1980; von Caemmerer, 2000) biochemical model of photosynthesis, light-saturated *A*_n_ at a given temperature can be limited by: (i) the maximum capacity of Rubisco carboxylation (*V*_cmax_) and internal CO_2_ concentration (*C*_i_); or (ii) the regeneration of ribulose 1,5-bisphosphate (RuBP) regeneration which relies on photosynthetic electron transport capacity (*J*) and irradiance. *A*_n_ can also be limited if triose phosphate use (TPU) restricts the recycling of phosphate within the chloroplast (Sharkey, 1985). *V*_cmax_, *J*, and TPU can be estimated by fitting the FvCB model (Farqhuar *et al.*, 1980) to plots of light-saturated *A*_n_ versus *C*_i_. Light-saturated rates of *A*_n_ respond to short- and long-term changes in growth environment temperature (Atkin and Tjoelker, 2003; Way and Yamori, 2014). The thermal response of light-saturated *A*_n_ to short-term changes in growth environment temperature is curvilinear—increasing with a rise in temperature at suboptimal temperatures to a maximum rate (*A*_max_) at the optimum temperature (*T*_opt_), then decreasing at temperatures beyond *T*_opt_. Longer term changes in the thermal environment cause either positive or detractive adjustments of light-saturated *A*_n_. Positive adjustments increase *A*_max_ and/or shift *T*_opt_ towards the new, higher growth temperature, indicating acclimation, whereas detractive adjustments reduce *A*_max_ and/or *T*_opt_ (Sage and Kubien, 2007; Way and Yamori, 2014; Posch *et al.*, 2019). Changes in the *A*_n_–temperature response are controlled by different factors, including the temperature sensitivities of *V*_cmax_ and *J* (Hikosaka *et al.*, 2005; Sage and Kubien, 2007; Lin *et al.*, 2012), with alterations in *V*_cmax_ reflecting changes in the abundance and/or activation state of Rubisco (Scafaro *et al.*, 2023). Another controlling factor, albeit indirectly, is stomatal conductance (*g*_s_). At high temperatures, stomatal closure reduces water loss when the atmospheric vapour pressure deficit (VPD) rises, but this simultaneously reduces CO_2_ uptake. In wheat, the *A*_n_–temperature response to elevated daily temperatures varies from positive acclimation to detractive adjustments (Yamasaki *et al.*, 2002; Cai *et al.*, 2018, 2020; Coast *et al*., 2021a). Importantly, information on thermal acclimation of wheat photosynthesis to specifically warm nights is scarce (but see Turnbull *et al.*, 2002 for work on a broadleaved tree species). It is likely that under night warming, wheat photosynthesis and acclimation capacity may be affected directly by constraining leaf biochemical components underlying photosynthesis (i.e. *V*_cmax_ and *J*), or indirectly by stomatal closure, or both. These suggestions remain untested.

The degree of physiological acclimation in leaves to warming varies substantially with the duration and intensity of warming, as well as the plant’s developmental stage. Leaves that develop under a new warming regime exhibit a higher degree of acclimation than pre-existing leaves that were formed under an earlier thermal regime and which then experience a sustained change in growth temperature (Loveys *et al.*, 2003; Yamori *et al.*, 2005; Rashid *et al.*, 2020; Slot *et al.*, 2021). Varying acclimation capacity with leaf development has also been shown in cold regimes (Hurry *et al.*, 2005; Campbell *et al.*, 2007). Limited reports exist about leaf development under warm conditions and the magnitude of photosynthetic acclimation in newly developed and pre-existing leaves. Determining these responses could be relevant in managing crop performance and useful in parameterizing carbon fluxes in Earth system models (Rogers *et al.* 2017).

*T*_crit_, a common measure of photosynthetic heat tolerance (Geange *et al.*, 2020; Lancaster and Humphreys, 2020), is estimated from temperature-dependent changes in minimum Chl *a* fluorescence (*F*_0_) (Schreiber *et al.*, 1975; Schreiber and Berry, 1977). *T*_crit_ correlates with the maximum temperature at which *A*_n_ approaches zero (Downton *et al*., 1984). Under warmer growth conditions, an increase in *T*_crit_ is considered evidence of acclimation and indicative of higher temperature tolerance. Acclimation of *T*_crit_ to warmer growth conditions has been shown in many non-crop, mostly woody species. For example, 62 Australian species acclimated seasonally with *T*_crit_, increasing on average from winter to summer by 0.34 °C per °C increase in growth temperature (Zhu *et al.*, 2018). Similar reports exist for Mediterranean and temperate European oak species (*Quercus* spp., Daas *et al.*, 2008), and at a global scale for 218 plant species spanning seven biomes (O’Sullivan *et al.*, 2017). Acclimation of *T*_crit_ is assumed to increase the upper thermal threshold of photosynthesis by relaxing the limits of photosynthetic electron transport. As such, the capacity to acclimate *T*_crit_ might underlie photosynthetic performance under warm nights. However, it is not known whether *T*_crit_ acclimates in response to warm nights.

Metabolites function as regulatory agents, compatible solutes, antioxidants, and reductants in adaptation to thermal stress. Warming alters the abundance of primary metabolites [sugars, sugar alcohols, organic acids, amino acids, or tricarboxylic acid (TCA) cycle intermediates] in leaves of stress-sensitive cereals (Glaubitz *et al.*, 2014; Impa *et al.*, 2019; Schaarschmidt *et al.*, 2020; Rashid *et al.*, 2021). Increases in the abundance of organic acids and TCA cycle components in heat-sensitive genotypes under heat stress often reflect impairment to CO_2_ assimilation, regulation of the TCA cycle, and amino acid biosynthesis, and these processes are central to plant photosynthesis. Deciphering changes in the metabolic phenotype of wheat in response to warm nights could aid our understanding of plant responses to climate change and provide input for developing adaptation tools for crop production in a warmer world.

To address some of the above issues, we compared four wheat genotypes (including a commercial Australian cultivar and an elite heat-tolerant genotype) under night temperatures of 15, 20, or 25 °C for different durations (5–7 d at anthesis or 9–13 weeks prior to and including anthesis). Our primary objectives were to: (i) quantify the acclimation capacity of photosynthesis and *T*_crit_ to warm nights; and (ii) assess changes in the wheat metabolic phenotype under warm nights. In addition, we set out to compare effects of warm nights on the temperature dependencies of photosynthetic parameters in newly developed versus pre-existing leaves. The results indicate that plants stressed by warm nights reduced photosynthetic performance via down-regulation of *J* even while exhibiting high *T*_crit_ (i.e. high PSII thermal stability). By contrast, tolerance of warm nights (in terms of photosynthesis) was marked by improved *A*_n_ linked to acclimation in *V*_cmax_, PSII thermal stability, and an increase in leaf metabolic signatures for monosaccharides.

## Materials and methods

To assess the extent to which photosynthetic capacity of pre-existing and newly developed leaves of wheat respond to warm nights, two experiments were conducted. In Experiment I, acclimation of *T*_crit_, instantaneous temperature response functions of photosynthetic capacity [light-saturated *A*_n_, *V*_cmax_, *J*_1500_ (*J* measured at a photosynthetic photon flux of 1500 µmol m^–2^ s^–1^), and TPU], and changes in the metabolite profile of plants that developed from seedling to anthesis (9–13 weeks of night warming) were compared at three night temperatures (15, 20, and 25 °C) and a common day temperature of 26 °C. These temperature treatments are reflective of conditions in some wheat-growing regions including parts of the USA (Hein *et al.*, 2020) and India (Bahuguna *et al.*, 2022). In Geraldton and Northampton on the northern tip of the West Australian wheatbelt, the mean minimum temperatures between October and December (i.e. during reproductive development to harvest) range from 12 °C to 17 °C. Similar temperatures, in controlled-environment settings, enable dissection of the physiological mechanism of tolerance and application of high-throughput phenotyping platforms to screen for stress tolerance; for example, see Wang *et al.* (2022) with control nights of 21 °C and high nights of 28 °C. Experiment II was a repetition of Experiment I except plants with mature (pre-existing) flag leaves which had grown under 15 °C nights were exposed to a shorter period of warming (5–7 nights at 20 °C and 25 °C environments) at anthesis. As such Experiment I focused on newly developed leaves and Experiment II on pre-existing leaves. For both experiments, all measurements were taken when plants were at the same developmental stage—anthesis (i.e. between Zadok scale ZS60 and ZS69; Zadoks *et al.*, 1974).

### Plant materials, management, and temperature treatments

Four wheat (*T. aestivum* L.) genotypes were used for this study: Mace (pedigree Wyalkatchem/Stylet/Wyalkatchem), a well-adapted, commercial, Australian cultivar; ACIAR09PBI C38-150C-DH9 (pedigree PBW343+L24+LR28/LANG; henceforth 1704), a heat-susceptible genotype; ACIAR09PBI C27-0C-0N-3N (pedigree DBW16/ANNUELLO; henceforth 1898), also a heat-susceptible genotype; and 8:ZW11 [pedigree D67.2/P66.270//AE.SQUARROSA(320)/3/CUNNINGHAM/4/VORB; henceforth 2254], a heat-tolerant elite genotype. Mace was a benchmark variety for yield in Western Australia where it was widely grown. It accounted for 66% of plantings between 2015 and 2016 (Zaicou-Kunesch *et al.*, 2017), although this reduced to 31% by 2018. Mace can cope with high temperatures (Bokshi *et al.*, 2022). The two heat-susceptible genotypes (1704 and 1898) exhibit low PSII thermal stability (i.e. low basal *T*_crit_) when grown under non-heat-stressed conditions but also the capacity to acclimate *T*_crit_ to warmer growth regimes in field conditions (Posch *et al.*, 2022b). Genotype 2254 was developed by the International Maize and Wheat Improvement Centre (CIMMYT) and, like Mace, it is considered heat tolerant (Posch *et al.*, 2022a). Unpublished yield data from field experiments conducted under heat stress conditions in Ciudad Obregon, Mexico and Narrabri, Australia by Professor Richard Trethowan of the University of Sydney, Australia, support the heat-tolerant classification of genotype 2254. These were chosen for their varied yield, and agronomic and physiological performance under heat stress conditions in fields across wheat-growing regions of Australia.

Seeds were germinated on moist filter papers in Petri dishes. One-week-old seedlings were sown into 6 litre plastic pots (one seedling per pot) filled with Martins mix (Martins Fertilizers Ltd, Yass, NSW Australia). The Martins mix was enriched with 4 g l^–1^ Osmocote® OSEX34 EXACT slow‐release fertilizer (Scotts Australia, Bella Vista, NSW, Australia) and treated at 63 °C for 1 h prior to filling pots. Potted seedlings were transferred into temperature-controlled growth chambers (Thermoline, Wetherhill Park, Australia) at the Controlled Environment Facilities of the Research School of Biology, The Australian National University, Canberra, Australia (ANU). Genotypes were arranged randomly in each chamber. Growth chambers were maintained at day/night temperatures of 26/15, 26/20. and 26/25 °C either throughout plant growth (lasting 9–13 weeks; Experiment I) or only at anthesis (lasting 5–7 d; Experiment II). For the latter conditions, day/night temperature prior to anthesis was maintained at 26/15 °C. In all growth chambers, relative humidity varied from 30% during the light period to 70% during the dark, and [CO_2_] was at ambient ~400 μmol mol^–1^ (38.4 Pa, considering a mean atmospheric pressure of 96 kPa at ANU). Lighting was supplied by 1000 W metal halide lamps (Multi-Vapor®; GE Lighting, Derrimut, Australia) producing photosynthetically active radiation at a plant height of 720–750 µmol m^–2^ s^–1^. A 12 h photothermal regime was maintained throughout plant growth. General plant management followed the established protocol of the Controlled Environment Facilities at ANU. Eight replicate plants of each genotype were assigned to each night temperature treatment.

### Determination of flag leaf photosynthetic heat tolerance

*T*_crit_ was estimated according to the method of Schreiber and Berry (1977) and recently applied by Zhu *et al.* (2018), Arnold *et al.* (2021), and Coast *et al.* (2021b). A detailed description of the method is given in Coast *et al.* (2021b); briefly, discs excised during the day from the middle section of detached dark-adapted leaves were exposed to a temperature ramp at a constant rate of 1 °C min^−1^ from 20 °C to 65 °C with simultaneous continuous measurement of *F*_0_ taken. *T*_crit_ was calculated as the intersection point of two regression lines extrapolated from the flat and steep portion of the *F*_0_–temperature response curve. *T*_crit_ was determined for all four genotypes in Experiment I, and for two genotypes (Mace and 1704) in Experiment II.

### Gas exchange measurements

Plants in growth chambers were moved into a temperature-controlled cabinet (Thermoline Model‐1175-SD-1SL, Thermoline Scientific, Smithfield, NSW, Australia) for all gas exchange measurements. Gas exchange measurements were conducted on intact flag leaves of the main tiller identified as the first tiller to reach anthesis. Five LI-COR portable photosynthesis systems (LI-6400XT, LI-COR Inc., Lincoln, NE, USA) were used for gas exchange measurements. The LI-COR units were fitted with 6 cm^2^ leaf chambers with a red–blue light source (6400-18 RGB Light Source, LI-COR). Leaves were exposed to saturating irradiance of 1500 μmol photons m^−2^ s^−1^ within the LI-COR leaf chamber, with both the LI-COR leaf chamber/block and the whole plant placed within the temperature-controlled cabinet. The LI-COR leaf chamber was initially set to 20 °C, reference line atmospheric [CO_2_] of 400 ppm, a flow rate of 500 μmol s^−1^, and relative humidity maintained between 40% and 75%. After flag leaves had been exposed to these conditions in the leaf chamber for at least 5 min and following equilibrium (stable readings for at least 1 min), *A*_n_ was determined. Thereafter photosynthetic [CO_2_] response curves (*A*:*C*_i_ curves) were generated, at a constant irradiance of 1500 μmol photons m^−2^ s^−1^, by varying the [CO_2_] inside the LI-COR leaf chambers as follows: 30, 50, 100, 150, 250, 400, 400, 600, 800, 1000, 1200, 1400, and 400 μmol mol^−1^. The *A*:*C*_i_ curves were repeated with the leaves exposed to measurement temperatures of 25, 30, 35, 40, and 50 °C. The temperature setting of the cabinet was adjusted to enable the LI-COR leaf chamber/block to achieve the desired measurement temperatures. Plants were kept well irrigated throughout the measurement period to avoid water stress. In both Experiment I and II, four replicate plants per night temperature treatment were used. All gas measurements were taken between 08.00 h and 17.00 h during weeks 9–13 for Experiment I or days 5–7 for Experiment II. The ranges (9–13 weeks, and 5–7 d) in period reflect differences in time to anthesis of the different genotypes.

### Modelling photosynthetic capacity

Model parameters for each growth and measurement temperature were estimated following the FvCB model and using the Plantecowrap package (Stinziano *et al.*, 2018) in the R computing environment (R Development Core Team, 2021). Plantecowrap eliminates potential bias associated with manually determining the concentration of intercellular CO_2_ (*C*_i_) where one limitation transitions to another, as is the case with the commonly used Sharkey Excel spreadsheet (Sharkey *et al.*, 2007), and allows for species-specific kinetic parameters to be pre-defined. The kinetic parameters used in modelling photosynthetic capacity for wheat were: mesophyll conductance at 25 °C (*g*_m_=5.5 μmol m^−2^ s^−1^ Pa^–1^); activation energy of mesophyll conductance (*E*_a_=47.65 kJ mol^–1^); apparent Michaelis–Menten constant for Rubisco carboxylation in 21% oxygen (*K*_air_=772 µmol mol^–1^); activation energy of *K*_c_ (93.72 kJ mol^–1^); photorespiratory CO_2_ compensation point or Gamma star at 25 °C (Γ*=37.74 µmol mol^–1^, equivalent to µbar bar^–1^); and Gamma star activation energy (24.42 kJ mol^–1^). The temperature responses of *V*_cmax_, *J*_1500_, and TPU were modelled using non-linear least squares fit of the Arrhenius temperature response function accounting for deactivation (Medlyn *et al.*, 2002; Kattge and Knorr, 2007). The deactivation energy (*E*_d_) was assumed to be 200 kJ mol^–1^; the activation energy (*E*_a_) and entropy factor (ΔS) were estimated from iterative fits of the model.

### Gas chromatography–mass spectrometry metabolite analysis

All leaf samples used for metabolite analysis were collected during the day (between 08.00 h and 17.00 h) and within an hour of completing gas exchange measurements. Metabolite extraction was conducted using a GC-MS procedure described in Rashid *et al.* (2021). Derivatization was performed using the MPS2 XL-Twister autosampler (Gerstel GmbH & Co. KG, Mülheim an der Ruhr, Germany), and metabolite samples were analysed on an Agilent GC/MSD system composed of an Agilent GC 6890N gas chromatograph (Agilent Technologies) fitted with a 7683B Automatic Liquid Sampler (Agilent Technologies) and 5975B Inert MSD quadrupole MS detector (Agilent Technologies). The gas chromatograph was fitted with a 0.25 mm (i.d.), 0.25 μm film thickness, 30 m Agilent FactorFour VF-5ms capillary column with a 10 m integrated guard column (Agilent Technologies). Raw GC-MS data were converted using the GC/MS Translator (ver 1.0, Agilent Technologies, Inc., Santa Clara, CA, USA) then processed (peak detection, retention time alignment, and relative quantitation) with MS-DIAL (ver 4.60; Tsugawa *et al.*, 2020). Metabolites were identified by comparing mass spectral features against available spectral libraries from the Golm Metabolome Database (Kopka *et al.*, 2004). Metabolite abundance values were normalized against the averaged signal of an internal standard ([^13^C_5_]valine) and the sample fresh mass, followed by weighting against the average measured signal across all samples for each compound.

### Statistical analysis

Data preparation, analysis, and visualization were performed with R (R Development Core Team, 2021) using the packages tidyverse (Wickham *et al.*, 2019), tidyr (Wickham, 2021), dplyr (Wickham *et al.*, 2021), ggplot2 (Wickham, 2016), FactoMineR (Lê *et al.*, 2008), factoextra (Kassambara and Mundt, 2020), ade4 (Chessel *et al.*, 2004; Dray and Dufour, 2007; Dray *et al.*, 2007; Bougeard and Dray, 2018; Thioulouse *et al.*, 2018), nls2 (Grothendieck, 2013), minpack.lm (Elzhov *et al.*, 2016), vegan (Oksanen *et al.*, 2020), lattice (Sarkar, 2008), latticeExtra (Sarkar and Andrews, 2022), car (Fox and Weisberg, 2019), AICcmodavg (Mazerolle, 2020), and agricolae (de Mendiburu, 2021).

Experiments I and II were analysed separately. Gas exchange data were checked for outliers prior to analysis. Differences in treatment means for *T*_crit_, gas exchange parameters (*A*_max_ and *g*_s_), and modelled photosynthetic capacities (*V*_cmax_ and *J*_1500_ at a standardized temperature of 25 °C; *T*_opt_ of *A*_n_, *V*_cmax_, *J*_1500_, and TPU; and *V*_cmax_, *J*_1500_, and TPU at *T*_opt_) were tested using ANOVA and treatments separated using Fisher’s LSD or Tukey’s HSD. Comparative regressions were used to test the effects of night temperature and genotype on the temperature response curves of *A*_n_, *V*_c_, *J*, and TPU. The regression models tested fits of a common line (the simplest model) and separate lines (the advanced model) for the genotype and night temperature. The simplest model assumed a common intercept and curvature, whereas the advanced model assumed differences in intercept or curvature, or both. *F*-tests were used to select the model with the best fit. The *A*_max_ (i.e. *A*_n_ at *T*_opt_) and *T*_opt_ of *A*_n_ were derived from equations of the second-order polynomials that best described the instantaneous *A*_n_–temperature relationships. Metabolite abundance data were analysed using principal component analysis (PCA) and a non-parametric, permutation tests-based, multivariate ANOVA (Anderson, 2001). This form of analysis generates test statistics analogous to Fisher’s *F*-ratio, and *P*-values are obtained using permutations.

## Results

**Fig. 1.**
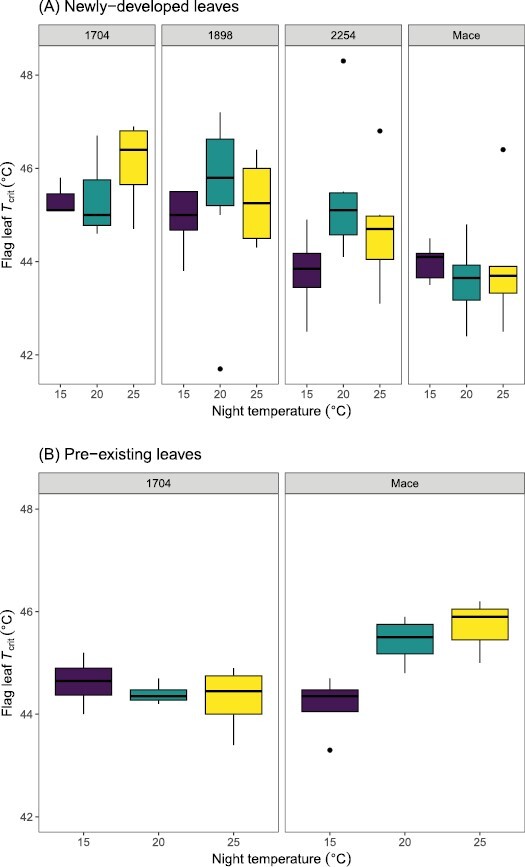
Photosynthetic high temperature tolerance (*T*_crit_) of newly developed leaves of four wheat genotypes (top panel) and pre-existing leaves of two wheat genotypes (bottom panel) at night temperatures of 15, 20, and 25 °C. The four genotypes were the heat-susceptible 1704 and 1898, and the heat-tolerant Mace and 2254. Means are of 3–8 plants.

*T*_crit_ of newly developed leaves (i.e. leaves that developed entirely under conditions where the prevailing night temperature was elevated) varied between genotypes (*P*<0.001) but did not significantly respond to night warming (Fig. 1A). Across night temperatures, mean *T*_crit_ of the heat-susceptible genotype 1704 (45.7 °C) was higher (*P*<0.05) than the *T*_crit_ of Mace (43.8 °C) and the elite genotype 2254 (44.6 °C), but not of genotype 1898 (45.2 °C, *P*=0.503). In pre-existing leaves (i.e. leaves that had developed under controlled-environment conditions and then exposed to 5–7 consecutive warm nights), responses of both genotypes to night warming differed: *T*_crit_ of Mace increased with warm nights whereas *T*_crit_ of 1704 did not significantly change, resulting in a significant genotype by night temperature interaction (*P*=0.008, Fig. 1B). Across night temperatures, Mace and 1704 differed in *T*_crit_ (*P*=0.015, for main effect of genotype). However, the effect of night temperature alone was not significant (*P*=0.107).

**Fig. 2.**
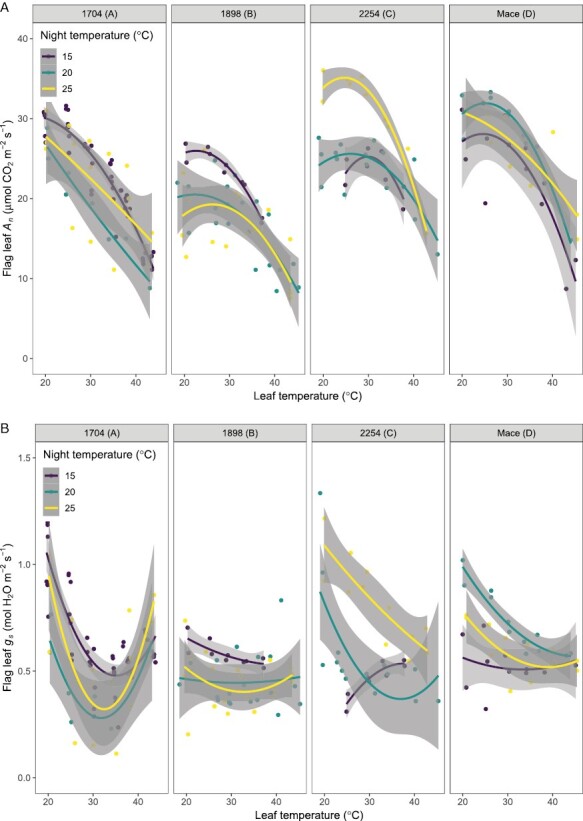
At ambient CO_2_: instantaneous temperature response curves of light-saturated net assimilation (*A*_n_; top panels) and stomatal conductance (*g*_s_; bottom panels) in wheat flag leaves that developed (newly developed) at a day temperature of 20 °C and night temperatures of 15, 20, or 25 °C. Individual panels are presented for the heat-susceptible genotypes 1704 (A) and 1898 (B), and the heat-tolerant genotypes 2254 (C) and Mace (D). For pre-existing leaves that only experienced night warming after spikes emerged and flowered, the instantaneous *A*_n_–temperature responses were unaffected by night warming (Supplementary Fig. S1). Lines are polynomial regression curves fitted to the data. The shaded regions indicate confidence intervals for the fitted polynomial curves. Means are of 2–4 plants.

When measured at ambient CO_2_, instantaneous temperature responses of light-saturated rates of *A*_n_ in newly developed wheat leaves responded to night warming (Fig. 2A; Table 1). The responses were characterized by maintaining or increasing the maximum rate of *A*_n_ (i.e. *A*_max_ at *T*_opt_) or the optimum temperature of *A*_n_ (*T*_opt_ of *A*_n_). The largest increase with night warming from 15 °C to 25 °C in *A*_max_ at *T*_opt_ and *T*_opt_ of *A*_n_ were in genotype 2254 and 1898, respectively. However, for genotype 1704, estimates of *A*_max_ at *T*_opt_ and *T*_opt_ of *A*_n_ for plants grown at 20 °C and 25 °C nights could not be determined due to the linear declines in *A*_n_ within the limited measurement temperature range studied. For genotype 1704 alone, comparison of regressions for the different night temperatures showed variation [*F*=65.07_(4, 68)_, *P*<0.001, and *R*^2^=0.79], but this was due to a lower *A*_n_–temperature response at 20 °C compared with both 15 °C and 25 °C nights. Stomatal conductance (*g*_s_) also responded to night warming, and was generally curvilinear with increasing measurement temperature (Fig. 2B). There was a clear interaction effect of genotype by night temperature on *g*_s_, with lower *g*_s_ for genotypes 1704 and 1898 under warmer nights, and the opposite for Mace and genotype 2254. The short-term leaf temperature response of *g*_s_ and *A*_n_ were curvilinear. Stomatal conductance was marked by an initial decline as temperature increased from 20 °C to ~30 °C, followed by an uptick at higher temperatures. There were minimal changes in photosynthesis for leaves that pre-existed when night temperatures were altered (Fig. 2C).

**Table 1.** Estimated optimum temperature (*T*_opt_) of light-saturated net assimilation at ambient CO_2_ (*A*_n_) in leaves of four wheat genotypes, and *A*_n_ at *T*_opt_ (i.e. maximum *A*_n_ or *A*_max_) derived from regression models of instantaneous temperature responses of *A*_n_

	Night temperature	Genotypes^*a*^			
		1704	1898	2254	Mace
Newly developed leaves^*b*^					
*T*_opt_ of *A*_n_	15	16.0	22.6	29.9	24.6
	20	---	22.3	26.0	25.2
	25	---	26.2	24.7	11.7
*A*_max_ (*A*_n_ at *T*_opt_)	15	30.5	26.0	25.2	28.1
	20	---	20.5	25.6	31.9
	25	---	19.3	35.1	31.6
Pre-existing leaves					
*T*_opt_ of *A*_n_	15–25	18.6	---	---	24.4
*A*_max_ (*A*_n_ at *T*_opt_)	15–25	21.0	---	---	23.4

^*a*^The heat-susceptible genotypes are 1704 and 1898, and the heat-tolerant genotypes are 2254 and Mace.

^*b*^The final models for newly developed leaves of each genotype were based on separate regression fits for the three different night temperatures, whereas for pre-existing leaves models were common regressions across the night temperature. --- The best fit was linear, *T*_opt_ could not be determined within the limits of temperature range tested.

**Fig. 3.**
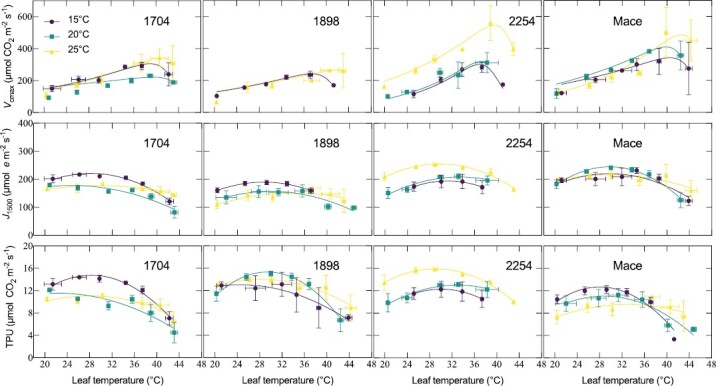
Temperature response curves of the maximum CO_2_ carboxylation capacity (*V*_cmax_), photosynthetic electron transport capacity (*J*_1500_), and triphosphate utilization (TPU) of leaves that developed (newly developed) at a common day temperature of 20 °C and night temperatures of 15, 20, or 25 °C for four wheat genotypes. The genotype name/ID are indicated in the panels for both the heat-susceptible genotypes 1704 (A) and 1898 (B), and the heat-tolerant genotypes 2254 (C) and Mace (D). *V*_cmax_ was iteratively fit with an Arrhenius equation with parameters given in Table 2. *J*_1500_ and TPU were fit with quadratic functions. Plots for pre-existing leaves of Mace and 1704 are presented in Supplementary Figure S2. Means are of 3–4 plants. Error bars represent the standard error of the mean.

*V*_cmax_ of newly developed leaves increased exponentially with rising leaf temperatures before peaking then declining at measurement temperatures above 36–47 °C for *V*_cmax_ and 29–34 °C for *J*_1500_ (Fig. 3; Table 2), which enabled a classical Arrhenius temperature response with a deactivation component to be fit. *T*_opt_ of *V*_cmax_ was unresponsive to night warming (*P*=0.112, Table 2) but modelled rates of *V*_cmax_ at *T*_opt_ increased significantly, between 24% and 43% (Fig. 3; Table 2). Below leaf temperatures of 36 °C, *V*_cmax_ and its response to temperature were similar between the genotypes and not affected by night temperature. Thus, the impact of night warming was most evident as an increase in Rubisco carboxylation capacity of leaves developed under the warmest nights.

**Table 2.** Mean estimates (±SD) of the optimum temperature (*T*_opt_) and maximum capacity of *V*_cmax_, *J*_1500_, and TPU in newly developed leaves of four wheat genotypes at three different night temperatures

Genotype^*a*^	Night temperature (°C)	*T*_opt_ of *V*_cmax_ (°C)	*V*_cmax_ at *T*_opt_ (μmol m^–2^ s^–1^)	*T*_opt_ of *J*_1500_ (°C)	*J*_1500_ at *T*_opt_ (μmol electrons m^–2^ s^–1^)	*T*_opt_ of TPU (°C)	TPU at *T*_opt_ (μmol m^-2^ s^-1^)
1704							
	15	38.1 ± 7.1	310 ± 32	29.4 ± 1.8	222 ± 8	29.6 ± 1.7	15 ± 2
	20	38.7 ± 0.2	217 ± 2	31.5	160	28.9	11
	25	41.6 ± 0.6	407 ± 28	32.8 ± 0.2	192 ± 17	32.6 ± 4.0	12 ± 4
1898							
	15	37.3 ± 3.4	242 ± 26	39.9 ± 1.6	195 ± 15	29.5 ± 2.0	13 ± 2
	20	---	---	29.2 ± 1.4	179 ± 35	29.0 ± 0.9	12 ± 1
	25	46.1 ± 4.8	346 ± 94	33.7 ± 6.1	181 ± 14	32.5 ± 7.0	11 ± 7
2254							
	15	39.3 ± 5.1	288 ± 32	29.2 ± 0.1	181 ± 37	28.5 ± 0.1	12 ± 3
	20	46.6	436 ± 2	34.1 ± 0.3	208 ± 20	32.6 ± 2.6	13 ± 2
	25	38.9 ± 2.3	498 ± 28	31.6 ± 1.5	259 ± 6	30.3 ± 2.3	16 ± 2
Mace							
	15	36.2 ± 6.2	326 ± 58	29.8 ± 3.4	213 ± 49	28.5 ± 4	13 ± 4
	20	39.1 ± 5.6	389 ± 33	30.8 ± 0.8	251 ± 23	30.1 ± 0.9	16 ± 1
	25	46.0 ± 9.8	569 ± 176	32.3 ± 6.5	225 ± 31	30.7 ± 6.9	14 ± 7
Levels of significance (*P*-values)							
	Genotype (G)	0.963	0.186	0.979	**0.024**	0.961	0.178
	Night temperature (NT)	0.112	**0.034**	0.156	0.851	0.330	0.942
	G x NT	0.568	0.796	0.854	**0.047**	0.922	0.078

^*a*^The heat-susceptible genotypes are 1704 and 1898, and the heat-tolerant genotypes are 2254 and Mace. --- The model fit could not be resolved. Highlighted in bold are *P*-values <0.05.

*J*_1500_ and TPU displayed a curvilinear response to leaf temperature (Fig. 3E, F). The genotype by night temperature effect on *J*_1500_ was significant (*P*=0.047, Table 2). This was due in part to *J*_1500_ decreasing with night warming for genotypes 1704 and 1898 but not being detrimentally affected by night warming in Mace and genotype 2254. *J*_1500_ at *T*_opt_ for Mace and 2254 increased by 5–30% with warmer nights (Table 2). By contrast, night warming reduced *J*_1500_ in 1704 and 1898 by 7–28% (Table 2). TPU was largely unresponsive to night warming and did not differ between genotypes. Like *T*_opt_ of *V*_cmax_, *T*_opt_ of *J*_1500_ and TPU were not significantly changed under warmer nights. However, *T*_opt_ values of photosynthetic capacity were high for *V*_cmax_ (range 37–47 °C) relative to *J*_1500_ (range 29–34 °C) and least for TPU (range 11–16 °C). As with net assimilation rates, photosynthetic capacity in terms of *V*_cmax_, *J*_1500_, and TPU were not influenced by night warming in pre-existing leaves of both genotypes (*P*>0.05, Supplementary Fig. S1; Supplementary Table S1).

**Fig. 4.**
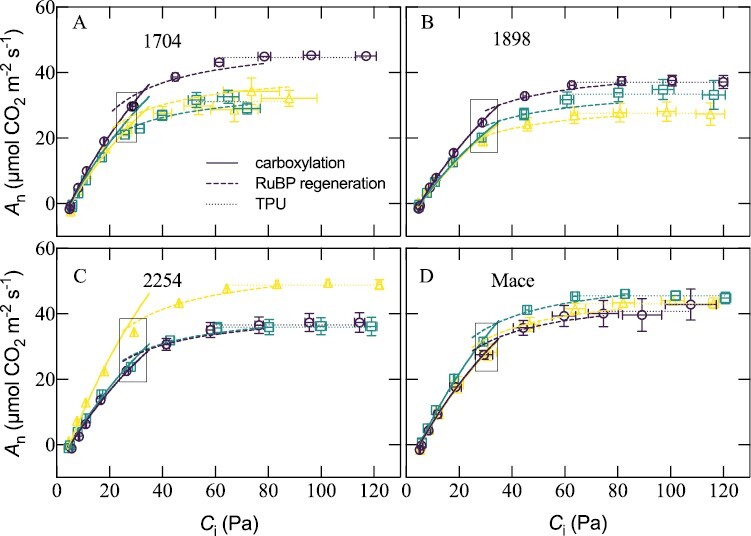
*A*–*C*_i_ curves and corresponding C_3_ photosynthesis model fits for newly developed leaves of wheat genotypes 1704 (A, heat-susceptible), 1898 (B, heat-susceptible), 2254 (C, heat-tolerant), and Mace (D, heat-tolerant). Plants were treated to a common day temperature of 20 °C and night temperatures of 15 (purple lines and shapes), 20 (green lines and shapes), or 25 °C (yellow lines and shapes). Solid, dashed, and dotted curves represent predicted carboxylation-limited *A*_n_ rates, RuBP regeneration-limited *A*_n_ rates, and triphosphate utilization- (TPU) limited *A*_n_ rates, respectively. The black outlined box encapsulates the values corresponding to ambient CO_2_ concentration of 400 μmol mol^–1^. Values are the means of pooled measurements at leaf temperatures of 25 °C and 30 °C and model parameters standardized to 28 °C. Means are of 4–6 plants.

To determine whether light-saturated *A*_n_ becomes RuBP limited in newly developed leaves, as suggested by the decline in *J*_1500_, *A*_n_ was plotted against *C*_i_ for the different night temperatures. The curvilinear responses were fitted using the modelled carboxylation and RuBP regeneration limitation rates (Fig. 4). At *C*_i_ corresponding to an ambient atmospheric CO_2_ concentration of 400 µmol mol^–1^, light-saturated *A*_n_ was reduced in 1704 and 1898 in response to a greater RuBP regeneration limitation at 20 °C and 25 °C nights (see black outlined boxes in Fig. 4). Genotypes 2254 and Mace either maintained or increased their RuBP regeneration capacity with night warming and consequently *A*_n_ at 400 µmol mol^–1^ across night treatments.

**Fig. 5.**
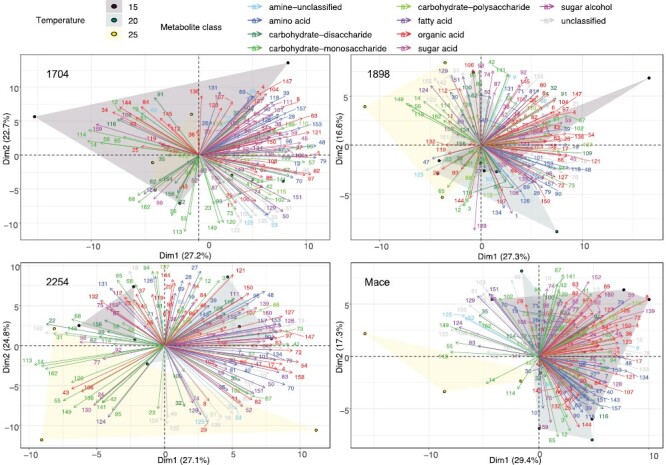
Principal component analysis (PCA) decomposition of metabolite responses in newly developed leaves of wheat genotypes to three different night temperature. The genotype name/ID are indicated in the panels for both the heat-susceptible genotypes 1704 and 1898, and the heat-tolerant genotypes 2254 and Mace. Metabolites are colour-coded by functional class. The direction and length of vector arrows provide information on the contribution each metabolite makes to dimensions 1 (Dim 1) and 2 (Dim 2), and the inter-relatedness of metabolites among themselves (opposing directions represent strong negative correlations while arrows of similar direction represent positive correlations), as well as in relation to night temperatures (the latter represented by blue, green, and red shaded areas). Arrow numbers correspond to individual metabolites which are listed in Supplementary Table S1, along with their relative abundance.

**Fig. 6.**
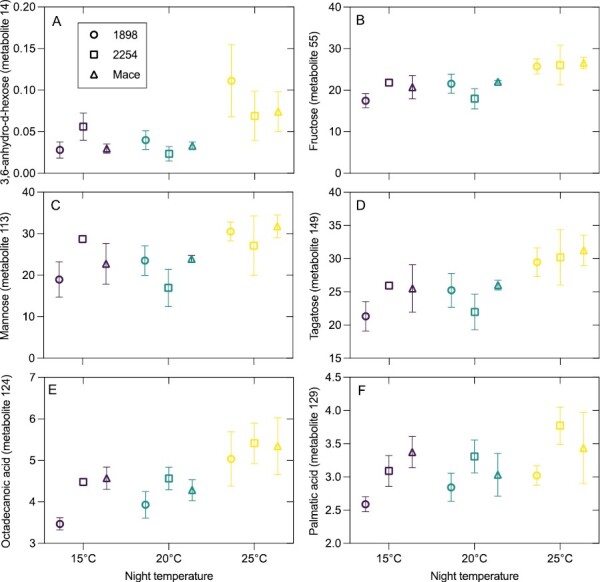
Individual metabolites that consistently responded positively to the warmest night of 25 °C in the genotypes 1898 (open circles; heat-susceptible), 2254 (open squares; heat-tolerant), and Mace (open triangles; heat tolerant). Metabolites presented are monosaccharides (A–D) and fatty acids (E, F). The genotype 1704 was excluded from this analysis as its metabolite profile showed no significant response to night temperature, evident in the PCA plots presented in Fig. 5. The metabolite numbers refer to their corresponding labels in the PCA plots.

Across the four genotypes and night temperatures of Experiment I, a total of 162 metabolites were identified in newly developed leaves. PCA determined relationships among individual metabolites and across treatments. Metabolites were assigned to classes as listed in Fig. 5 (metabolite relative abundance and classification are presented in Supplementary Table S2). The response of metabolites generally followed a similar pattern for a particular class, and often a class responded independently of other classes. Most notably, organic acids and amino acids clustered in the right half of the PCA (positive Dim1), while many carbohydrates clustered in the opposite left half. Thus, organic and amino acid abundance were in general positively related to one another and negatively correlated with many carbohydrates. In terms of night treatment, there was a shift to a more distinct separation of metabolite abundance in genotypes that were more physiologically responsive and tolerant to the warmer nights (Fig. 5). For example, the most sensitive and non-responsive genotype 1704 had little separation of metabolite profiles among night temperatures—evident in overlapping night temperature-dependent metabolite distribution clouds in Fig. 5. By contrast, the two most heat-responsive and tolerant genotypes (Mace and 2254) had a distinct special separation of the 25 °C night treatment metabolites, represented by special separation at negative Dim1 and Dim2 values (i.e. the lower left corner of PCA graphs). Genotype 1898 had intermediate separation of metabolite profiles in response to night warming, shifting to negative Dim1 values. Interestingly, many of the same metabolites positively correlated with warmer nights for the three genotypes (1898, 2254, and Mace) that responded to warmer nights (Fig. 6). Of these metabolites that responded positively, most were monosaccharides, namely 3,6-anhydro-d-hexose, fructose, mannose, mannose-6-phosphate, and tagatose (metabolites 14, 55, 113, 114, and 149, respectively). The other metabolites that consistently responded to the warmest night of 25 °C were the saturated 18:0 and 16:0 fatty acids octadecanoic acid and palmitic acid (metabolites 124 and 129).

## Discussion

In this study, we showed that wheat photosynthesis and acclimation capacity are directly affected by night warming, with varying genotypic responses and differences between newly developed and pre-existing leaves. For most genotypes, metabolites for monosaccharides and saturated fatty acids had the strongest positive correlation to warmer nights. Osmotic regulation and changes in fatty acid saturation with warming, which is consistent with changing membrane structural integrity (Rudolph and Goins, 1991; Harishchandra *et al.*, 2010; Marček *et al.*, 2019; Siddiqui *et al.*, 2020), may be linked to the heat tolerance potential of wheat to night warming. Difference between newly developed and pre-existing leaves were consistent with previous physiological reports of plants demonstrating a far greater acclimation potential in tissue that developed under a particular thermal regime rather than simply experiencing thermal change post-development (Armstrong *et al.*, 2006; Campbell *et al.*, 2007; Rashid *et al.*, 2020). However, we note that our results are based on a small number of genotypes, with a focus on photosynthesis-related traits measured at a single growth stage (anthesis) in controlled-environment conditions. Thus, possible conclusions about mechanisms of high night temperature tolerance are limited and deserve further attention in future studies.

### Heat-tolerant wheat genotypes can sustain *V*_cmax_ at higher leaf temperatures when grown at warmer nights

Two heat-tolerant genotypes (Mace, a locally adapted commercial cultivar; and 2254, an elite heat-tolerant genotype) were able to either maintain or increase CO_2_ fixing capacity at hotter leaf temperatures in response to warmer nights (Fig. 3). This was most evident in the significantly greater *V*_cmax_ reached at its *T*_opt_ when grown under warmer nights (Table 2; Fig. 3). An increase in photosynthetic capacity through an increase in *V*_cmax_ and its *T*_opt_, in response to both day and night warming, is evident across a range of plant species (Kattge and Knorr, 2007; Smith and Dukes, 2017). The fact that we observed these acclimation responses in wheat genotypes due to increases in night temperature alone is interesting considering that the night temperature was not expected to have a direct influence on photosynthesis due to the temporal separation of the night warming from photosynthesis in C_3_ species. Night-time respiration is more likely to be a major contributor to acclimation than photosynthesis. Although we have focused on photosynthesis and not respiration, we expect that night warming will alter the ratio of dark respiration to *A*_n_, similar to previous studies (Turnbull *et al.*, 2002; Bahuguna *et al.*, 2017; Impa *et al.*, 2019).

The stable to higher *V*_cmax_ at hotter leaf temperature in 2254 and Mace with warming nights was probably a result of more active Rubisco rather than a change in its abundance or kinetics (Scafaro *et al.*, 2023). It is well established, including in wheat, that a proportion of Rubisco is inactive, and this inactivity increases with leaf temperature (Law and Crafts-Brandner, 1999; Sharwood *et al.*, 2016; Perdomo *et al.*, 2017). The decline in *V*_cmax_ at the hotter leaf temperatures is consistent with Rubisco becoming inactive. Declines in Rubisco activity with heat are not due to Rubisco *per se* which is a thermally stable protein (Salvucci *et al.*, 2001), but rather due to the heat-labile nature of Rubisco activase (Rca), its accessory protein (Salvucci and Crafts-Brandner, 2004). The stable to higher *V*_cmax_ of the heat-tolerant genotypes at hot leaf temperatures—and only when grown at warmer nights—suggests that night warming is inducing changes in Rca and its control over Rubisco. Recently, a thermally stable isoform of Rca (Rca1β) was identified in wheat, and its expression and abundance increase with exposure of wheat to heat stress (Scafaro *et al.*, 2019; Degen *et al.*, 2021; Perdomo *et al.*, 2021). Night warming-induced expression of Rca1β in 2254 and Mace but not in 1704 and 1898 would confirm this hypothesis.

The shift in *V*_cmax_ with night temperature could potentially be due to temperature-dependent shifts in the kinetic properties of Rubisco. However, there are limited indications that Rubisco kinetics may alter in response to growth conditions. One example is spinach (*Spinacia oleracea*), when grown at the hotter day/night temperature of 30/25 °C compared with 15/10 °C (Yamori *et al.*, 2006). A small but significant increase in the thermal stability of spinach Rubisco implied acclimation-induced alterations in Rubisco functionality. A wider study, which included wheat, demonstrated that Rubisco properties such as its CO_2_ substrate affinity can differ depending on growth temperature (Orr *et al.*, 2016). More studies are needed to conclusively determine if Rubisco isoforms and associated kinetics within a species can shift in response to growth temperature. What is most unlikely is that night warming increased Rubisco abundance, as this would manifest as an increase in *V*_cmax_ across all measured leaf temperatures, but night warming did not influence *V*_cmax_ at leaf temperatures below 36 °C (Fig. 3). Furthermore, growth of wheat under both hotter days and nights does not appear to change Rubisco abundance (Perdomo *et al.*, 2017).

### CO_2_ conductance and electron transport limit photosynthesis in the heat-susceptible genotypes with night warming

Newly developed leaves of the genotype 1898 and the heat-susceptible genotype 1704 (known to be susceptible in terms of yield and growth in the field under hotter growth conditions) exhibited reduced photosynthesis rates when night temperature was warmer than 15 °C. The detrimental effects of night warming on the photosynthetic activity could be attributed to a decline in CO_2_ substrate availability, electron transport capacity, and TPU under the warmer nights (Figs 2–4). Declines in *g*_s_ with night warming are associated with a decline in the *C*_i_/*C*_a_ ratio. A decline in *C*_i_/*C*_a_ (as a result of decreased *g*_s_) without an increase in *A*_n_ is a strong indication of water stress and CO_2_ substrate limitations on photosynthetic rates (Morison and Gifford, 1983; Condon *et al.*, 2004). Thus, it is likely that these wheat genotypes had a sensitive response to perceived limitations in water availability triggered by night warming, or adopted reduced *g*_s_ by increasing abscisic acid in direct response to heat stress (Rodriguez and Davies, 1982; Li *et al.*, 2020). It may also have been due to the link between temperature or VPD and stomatal conductance. For the well-adapted Mace and heat-tolerant elite genotype 2254, the strong coupling of *g*_s_ with *A*_n_ (Lawson *et al.*, 2011), and other mechanisms linked to water status (e.g. increase in xylem and mesophyll hydraulic conductance; Urban *et al.*, 2017a), would explain the parallel increases in *g*_s_ and *A*_n_ at higher nocturnal temperature. The assumption of a fixed *g*_s_–*A*_n_ relationship is central to many models of stomatal control of photosynthesis at different scales: leaf, plant, ecosystem, and global circulation models (Farquhar and Wong, 1984; Leuning 1995; Buckley *et al.*, 2003; Verhoef and Egea, 2014). However, this *g*_s_–*A*_n_ relationship can be decoupled under extreme conditions such as heatwaves (Ameye *et al.*, 2012; von Caemmerer and Evans, 2015) or at extremely high leaf temperatures (Urban *et al.*, 2017b).

Night warming above 15 °C reduced the photosynthetic electron capacity and/or the *T*_opt_ of *J*_1500_ of genotypes 1704 and 1898 (Fig. 3; Table 2). This reduction in *J*_1500_ led to a modelled RuBP regeneration limitation in *A*_n_ at current ambient CO_2_ concentrations (Fig. 4). Despite the reductions in *J*_1500_ and/or the *T*_opt_ of *J*_1500_ with night warming, PSII was found to be thermally stable (higher *T*_crit_) than the heat-tolerant Mace and 2254 (Fig. 1A). The mismatch between *T*_crit_ and *J*_1500_ demonstrates that the upper temperature limit of electron transport is not necessarily aligned with susceptibility of electron transport rates under the prevailing growth temperature. In other words, photosynthesis of 1898 and 1704 may have an ability to withstand a more severe heat shock despite being more susceptible to loss of function under milder but more sustained heating. Genotype 2254 was able to increase *J*_1500_ and had relatively larger increases in *T*_crit_ with night warming than 1704 and 1898, possibly meaning that the extent of change in *T*_crit_ is more informative than the upper limit of *T*_crit_ when assessing the potential for electron capacity to adjust to temperature. Pre-existing leaves of Mace, but not 1704, responded strongly to warmer nights. This suggests a greater role for older leaves in heat stress resilience, even though these leaves are assumed to have less capacity to acclimate to high temperatures.

In contrast to *V*_cmax_, the response of *J*_1500_ and TPU to leaf measuring temperatures did not follow an expected exponential rise that could be fit with an Arrhenius equation (Fig. 3). A similar finding has previously been observed in wheat for *J* (Silva-Perez *et al.*, 2017). This is likely to be linked to *V*_cmax_ being driven by the kinetics of Rubisco enzyme and its exponential response to temperature (apart from the aforementioned decline at hotter leaf temperatures due to Rca loss of function). *J*_1500_ is set by more complex protein- and membrane-dependent interactions, some of which may not respond as dynamically to short-term rises in leaf temperature. TPU, on the other hand, largely comes into play at high CO_2_ levels, beyond the ambient conditions under which these experiments were conducted.

### Monosaccharide and saturated fatty acid accumulation are key responses of metabolite to warm nights

The most striking change in metabolites was a build-up of monosaccharide soluble sugars in newly developed leaves of the more heat-tolerant genotypes when grown at warmer nights. The build-up of monosaccharides may be related to maintenance of the osmotic potential of cells (Marček *et al.*, 2019) and regulation of stomatal conductance, which is also linked to photosynthesis (Flütsch *et al.*, 2020). Soluble sugars are potent contributors to cell osmotic potential and regulation (Moinuddin *et al.*, 2005; Blum, 2017). It is noted that osmolyte adjustments contribute to drought and heat stress avoidance in wheat (Blum and Pnuel, 1990). Specific to night warming, the disaccharide trehalose accumulated in response to night warming in wheat spike tissue (Impa *et al.*, 2019). Thus, the accumulation of soluble sugars in the heat-responsive genotypes but not in 1704 under warmer nights may have contributed to greater osmotic protection during warmer nights. The assumed decline in osmotic potential of the heat-responsive 2254 and Mace leaves may have contributed to its increase in *g*_s_ with warmer nights, as opposed to 1704 which had a reduction in *g*_s_. Solutes, including soluble sugars, have also been shown to provide protection to membrane structural integrity during stress through greater hydration of the lipid surfaces (Rudolph and Goins, 1991; Harishchandra *et al.*, 2010). The higher abundance of simple soluble carbohydrates together with night warming may have contributed to the maintenance of electron transport (maintained and even increased *J*_1500_) and ability to acclimate *T*_crit_ to a greater extent than 1704. In agreement with our postulation that osmolytes are protecting *J*_1500_ in 2254 and Mace but not 1704, supplementing *Populus tremula* L. leaves with increasing amounts of the monosaccharide sucrose substantially reduced leaf osmotic potential and led to an increase in stability of photosynthetic electron transport at higher *T*_leaf_ (Hüve *et al.*, 2006). That we observed a consistent increase in the saturated fatty acids octadecanoic acid and palmitic acid in the more photosynthetically heat-tolerant genotypes with night warming is a further indication of membranes adjusting to be more stable in response to warmer temperatures. Saturation of fatty acids in membranes is known to be a key heat tolerance response of plants (Larkindale *et al.*, 2005; Zhu *et al.*, 2018). Our results suggest that soluble sugar and saturated fatty acid contents in wheat leaves are key metabolites linking night warming and alterations in day processes. The connection between night temperature, carbohydrate and saturated fatty acid accumulation, and CO_2_ conductance and electron transport capacity needs further exploration. Having identified a specific heat tolerance metabolic profile, high-throughput metabolic approaches such as hyperspectral imaging could be used to build models to predict this metabolic profile as has been achieved for amino acids in maize (Shu *et al.*, 2022). These high-throughput measures could then be deployed by wheat populations through both ground and aerial sensing applications to determine heat tolerance.

### Conclusions

Night warming has significant implications on the photosynthetic performance of wheat leaves that have developed under the prevailing night temperature. The heat-tolerant genotype (2254 and Mace) had higher light-saturated *A*_n_ when grown at warmer nights, in contrast to the other genotypes (1704 and 1898) which had reduced *A*_n_. These night temperature-dependent differences in *A*_n_ between genotypes of wheat could in part be attributed to how night warming influenced photosynthetic capacity and CO_2_ conductance. The thermally tolerant genotypes had a more stable *V*_cmax_ at higher leaf temperatures when grown at warmer nights, which is likely to be due to night-dependent alteration to the temperature sensitivity of the activation state of Rubisco. The thermally susceptible genotypes had reduced *g*_s_ and *J*_1500_ when grown at warmer nights, which accounts for the corresponding reduction in *A*_n_. The stability of electron transport and CO_2_ conductance in the heat-tolerant genotypes with night warming may be linked to the greater accumulation of monosaccharides and saturated fatty acids in its leaves, balancing osmotic pressure alterations and cell membrane integrity on exposure to warm nights. The lack of a night warming response in pre-existing leaves, as well as differences between the two wheat genotypes, demonstrates the divergence of strategies needed to improve wheat performance under future climate trends. Improving CO_2_ conductance and photosynthetic capacity may be beneficial in some genotypes and potentially achieved through altering monosaccharide and saturated fatty acid contents of cells. In other genotypes with no detrimental impact of night warming on photosynthetic performance (exemplified by the heat-tolerant 2254 and Mace), it might be best to focus on sink tissue limitations and other physiological processes detrimentally affected by heat and not photosynthetic capacity. An important physiological process that clearly deserves investigating is mitochondrial respiration in the dark.

## Supplementary data

The following supplementary data are available at *JXB* online.

Fig. S1. Instantaneous temperature response curves of light-saturated net assimilation (*A*_n_) and stomatal conductance (*g*_s_) in pre-existing flag leaves of four wheat genotypes at ambient CO_2_.

Fig. S2. Temperature response curves of the maximum CO_2_ carboxylation capacity (*V*_cmax_), photosynthetic electron transport capacity (*J*_1500_), and triphosphate utilization (TPU) of pre-existing wheat leaves at night temperatures of 15, 20, or 25 °C.

Table S1. Mean estimates (±SD) of the optimum temperature (*T*_opt_) and maximum capacity of *V*_cmax_, *J*_1500_, and TPU in pre-existing leaves of two wheat genotypes at three different night temperatures.

Table S2. Metabolite relative abundance and classification.

erad437_suppl_Supplementary_Figures_S1-S2_Tables_S1

erad437_suppl_Supplementary_Tables_S2

## Data Availability

All primary data to support the findings of this study are openly available in the Dryad Digital Repository at doi: 10.5061/dryad.fqz612jx7 (Coast *et al.*, 2023).
