# Supplementary material for: Photosynthesis in newly developed leaves of heat-tolerant wheat acclimates to long-term nocturnal warming
Source: J Exp Bot. 2023 Nov 4;75(3):962–78. doi: 10.1093/jxb/erad437 (PMC10837020; doi:10.1093/jxb/erad437)
Supplement: erad437_suppl_Supplementary_Figures_S1-S2_Tables_S1 [file erad437_suppl_supplementary_figures_s1-s2_tables_s1.pdf]

SUPPLEMENTARY DATA

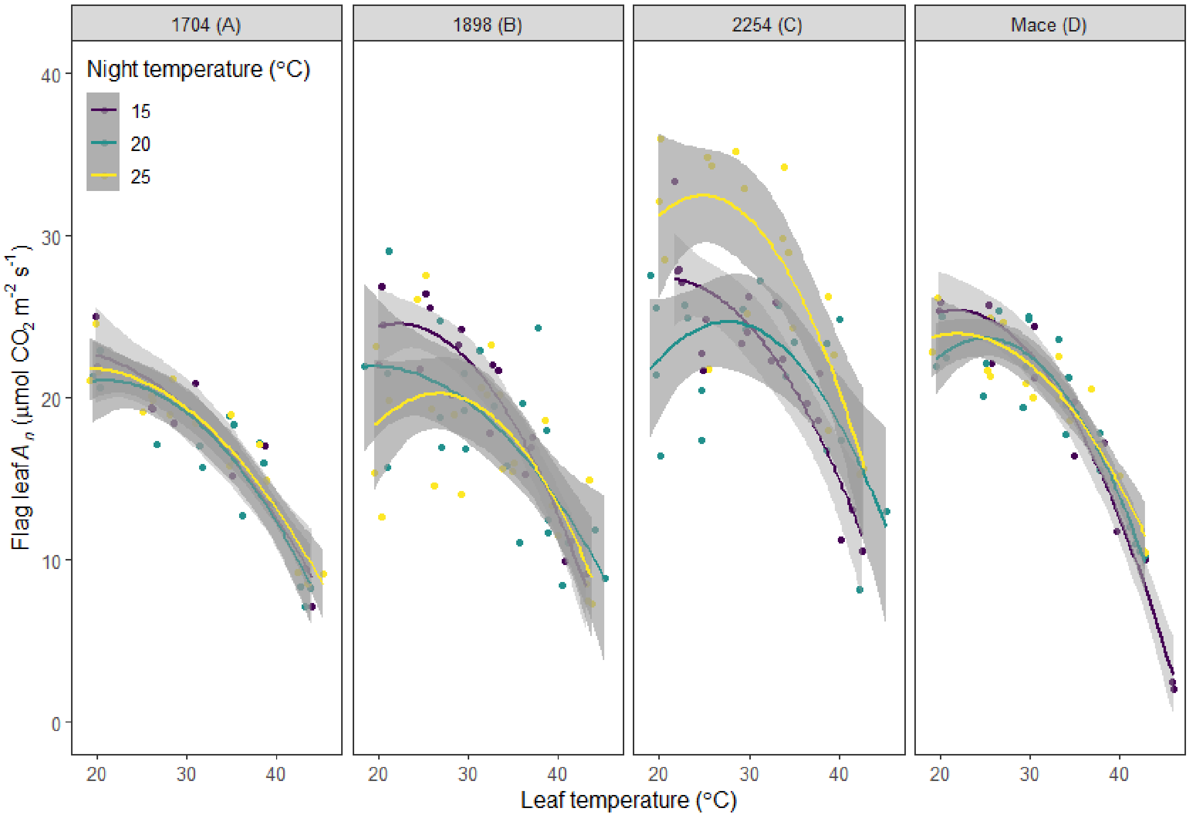

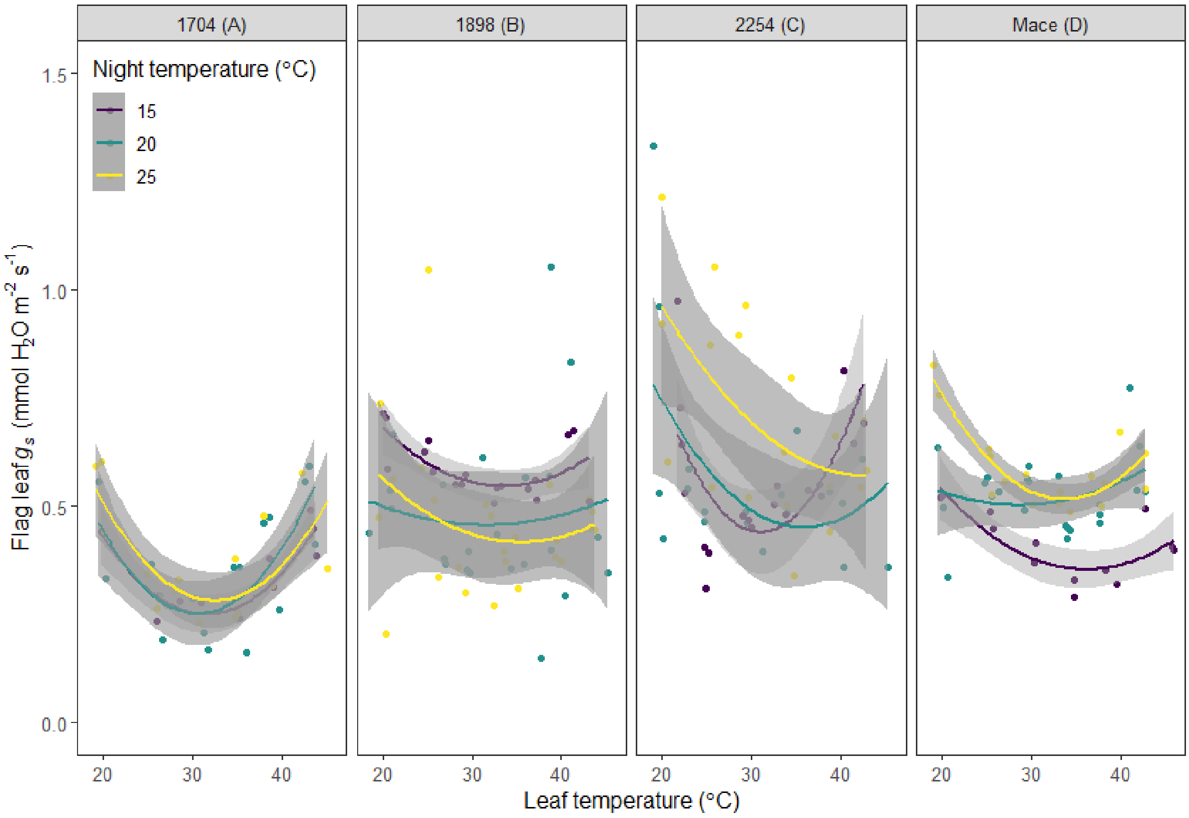

Figure S1. At ambient CO_2: Instantaneous temperature response curves of light-saturated net assimilation (A_n), and stomatal conductance (g_s) in pre-existing flag leaves of four wheat genotypes. The four genotypes were the heat-susceptible 1704 and 1898, and the heat-tolerant Mace and 2254. These were grown at a common day temperature of 20 °C and treated to night temperatures of 15, 20, or 25 °C (panels A-C) after spikes emerged and flowered.

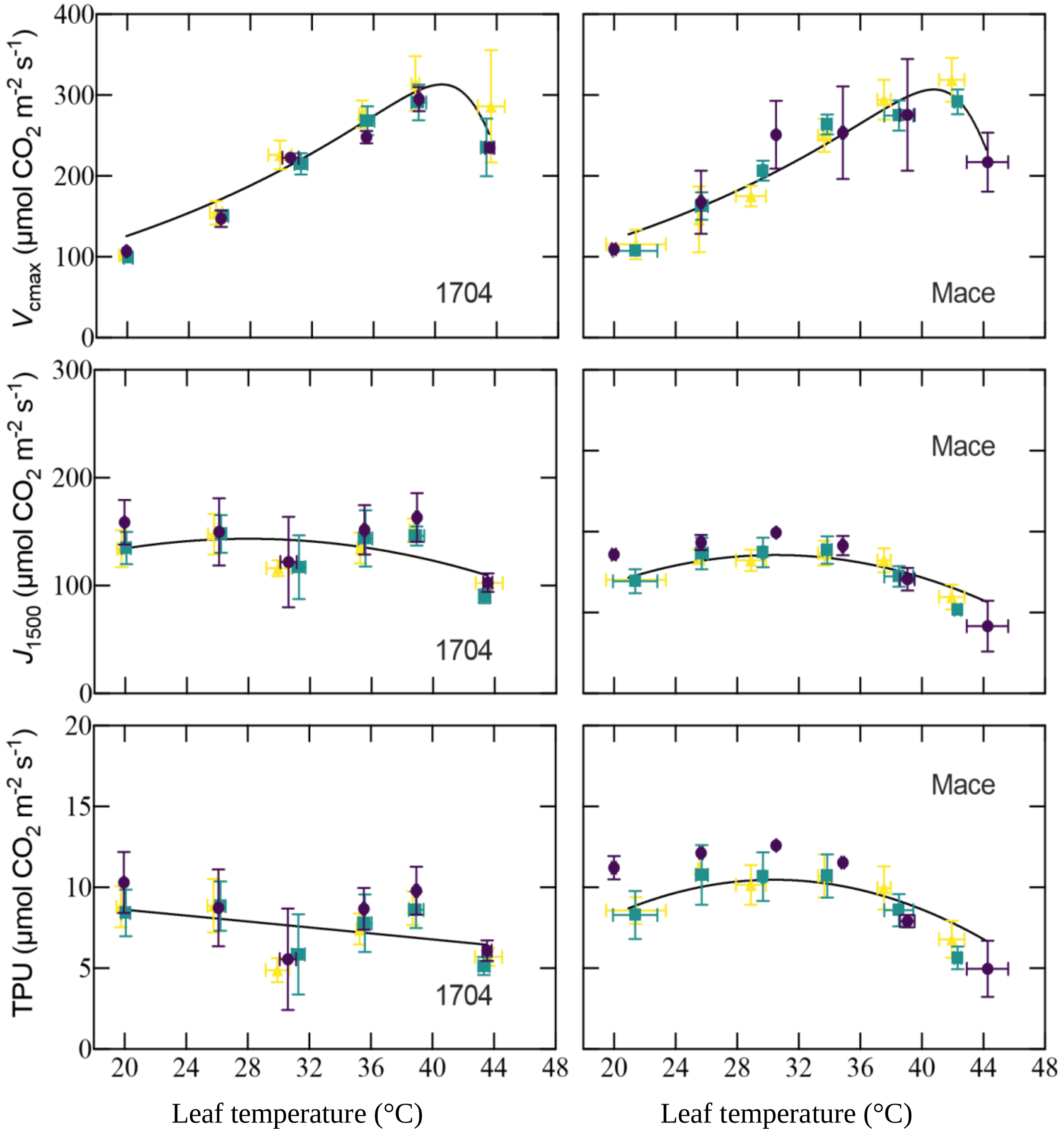

Figure S2. Temperature response curves of the maximum CO_2 carboxylation capacity (V_{cmax}), photosynthetic electron transport capacity (J_{1500}) and triphosphate utilisation (TPU) of pre-existing leaves treated to a common day temperature of 20°C and night temperatures of 15 (purple lines and shapes), 20 (green lines and shapes), or 25 (yellow lines and shapes) °C in wheat. The wheat genotypes 1704 (heat susceptible) and Mace (heat tolerant) are indicated on panels. V_{cmax} was iteratively fit with an Arrhenius equation with parameters given in Supplementary Table S1. J_{1500} and TPU were fit with quadratic functions, where possible. Means are of 3 to 4 plants. Error bars represent the standard error of the mean.

Supplementary Table S1. Mean estimates (± standard deviation) of the optimum temperature (T_{opt}) and maximum capacity of V_{cmax}, J_{1500} and TPU in pre-existing leaves of two wheat genotypes at three different night temperatures.

Genotype[1]	Night temperature (°C)	T_{opt} of V_{cmax} (°C)	V_{cmax} at T_{opt} (μmol m^{-2} s^{-1})	T_{opt} of J_{1500} (°C)	J_{1500} at T_{opt} (μmol electrons m^{-2} s^{-1})	T_{opt} of TPU (°C)	TPU at T_{opt} (μmol m^{-2} s^{-1})
1704							
	15	38.8±0.1	280±3	29.7±0.2	151±45	---	---
	20	38.5±2.6	285±41	31.0±0.2	168±32	23.7±5.8	10±3
	25	39.3±4.3	331±86	32.5±3.5	134±11	29.3	6
Mace							
	15	38.0±2.4	280±84	30.2±0.8	195±18	29.3±0.4	13±1
	20	37.7±2.3	301±24	31.9±0.9	181±37	31.4±1.0	11±3
	25	40.9±5.7	309±1	32.5±0.7	175±28	31.8±0.5	11±3
Levels of significance (*P*-values)							
	Genotype (G)	0.909	0.901	0.393	0.095	0.**009**	0.168
	Night temperature (NT)	0.639	0.509	0.063	0.643	0.284	0.622
	G x NT	0.592	0.851	0.864	0.710	0.159	0.398

[1]The heat-susceptible genotype 1704 and the heat-tolerant genotype Mace. --- model fit was either poor or could not estimate value. P <0.05 are highlighted in bold.
